# Reducing shame in a game that predicts HIV risk reduction for young adult men who have sex with men: a randomized trial delivered nationally over the web

**DOI:** 10.7448/IAS.16.3.18716

**Published:** 2013-11-13

**Authors:** John L Christensen, Lynn Carol Miller, Paul Robert Appleby, Charisse Corsbie-Massay, Carlos Gustavo Godoy, Stacy C Marsella, Stephen J Read

**Affiliations:** 1 Department of Communication and Center for Health, Intervention, & Prevention, University of Connecticut, Storrs, CT; 2 Annenberg School for Communication and Journalism, University of Southern California, Los Angeles, CA; 3 Department of Paediatrics (CHLA), Keck School of Medicine, University of Southern California, Los Angeles, CA; 4 S. I. Newhouse School of Public Communication, Syracuse University, Syracuse, NY; 5 Institute for Creative Technologies, University of Southern California, Playa Vista, CA; 6 Department of Psychology, University of Southern California, Los Angeles, CA

**Keywords:** stigma, shame, intervention, serious games, SOLVE, HIV, AIDS, sexual risk-taking, men who have sex with men (MSM)

## Abstract

**Introduction:**

Men who have sex with men (MSM) often face socially sanctioned disapproval of sexual deviance from the heterosexual “normal.” Such *sexual stigma* can be internalized producing a painful affective state (i.e., shame). Although shame (e.g., addiction) can predict risk-taking (e.g., alcohol abuse), sexual shame's link to sexual risk-taking is unclear. Socially Optimized Learning in Virtual Environments (SOLVE) was designed to reduce MSM's sexual shame, but whether it does so, and if that reduction predicts HIV risk reduction, is unclear. To test if at baseline, MSM's reported past unprotected anal intercourse (UAI) is related to shame; MSM's exposure to SOLVE compared to a wait-list control (WLC) condition reduces MSM's shame; and shame-reduction mediates the link between WLC condition and UAI risk reduction.

**Methods:**

HIV-negative, self-identified African American, Latino or White MSM, aged 18–24 years, who had had UAI with a non-primary/casual partner in the past three months were recruited for a national online study. Eligible MSM were computer randomized to either WLC or a web-delivered SOLVE. Retained MSM completed baseline measures (e.g., UAI in the past three months; current level of shame) and, in the SOLVE group, viewed at least one level of the game. At the end of the first session, shame was measured again. MSM completed follow-up UAI measures three months later. All data from 921 retained MSM (WLC condition, 484; SOLVE condition, 437) were analyzed, with missing data multiply imputed.

**Results:**

At baseline, MSM reporting more risky sexual behaviour reported more shame (*r*_s_=0.21; *p*<0.001). MSM in the SOLVE intervention reported more shame reduction (*M=*−0.08) than MSM in the control condition (*M*=0.07; *t*(919)=4.24; *p*<0.001). As predicted, the indirect effect was significant (point estimate −0.10, 95% bias-corrected CI [−0.01 to −0.23] such that participants in the SOLVE treatment condition reported greater reductions in shame, which in turn predicted reductions in risky sexual behaviour at follow-up. The direct effect, however, was not significant.

**Conclusions:**

SOLVE is the first intervention to: (1) significantly reduce shame for MSM; and (2) demonstrate that shame-reduction, due to an intervention, is predictive of risk (UAI) reduction over time.

## Introduction

From 2006 to 2009, there was a 21% increase in HIV incidence among those aged 13–29, largely due to a 34% increase in young men who have sex with men (MSM) [1]. Despite successes [2], HIV prevention for MSM falls short, perhaps because interventions to reduce unprotected anal intercourse (UAI) do not address the “discrimination and homophobia,” – and stigma – that “fuel the HIV epidemic in gay and bisexual men” [3]. S*exual stigma* has been defined as “the negative regard, inferior status, and relative powerlessness that society collectively accords to any non-heterosexual behaviour, identity, relationship, or community” [4].

MSM can internalize sexual stigma [4], producing shame [5–7]. Shame may operate quite differently from other painful affective states, such as fear, that can impact risk-taking [8]. Shame provides immediate feedback (i.e., punishment) regarding whether one's past, current or anticipated future (sexual) behaviour is in line with one's moral standards [9]. Shame has been differentiated from guilt empirically in that shame results from seeing one's situation as unchangeable (stable) and attributable to the individual as a whole (global) whereas guilt is caused by unstable, non-global attributions [10, 11]. When stable desires (e.g., for other men) and moral standards (e.g., one should not desire sex with a man) conflict and neither seems changeable, one may perceive the self as responsible for uncontrollable outcomes, and the global self may be devalued [5], resulting in a state of shame [9, 11, 12]. Shame and guilt can each be experimentally activated (e.g., imagining a given situation) and produce differential brain patterns as assessed by functional magnetic resonance imaging [13].

States of shame, but not guilt, have been linked to a variety of negative health outcomes [9]. For example, recovering alcoholics’ displays of shame (e.g., humped shoulders) when describing their last drink predicted subsequent relapse severity [14]. States of shame differ from trait-like constructs (e.g., internalized homophobia that inconsistently predicts sexual risk-taking [15]) in that they involve *specific situational contexts* (e.g., before an attractive partner). For example, participants given misleading feedback – suggesting their responses conflicted with their self-standards – felt shame [16]. If conflicting standards and responses produce shame, perhaps reducing perceived conflict might reduce shame. Conflict between some MSM's self-standards (e.g., having sex with men is wrong) and their actual reactions (e.g., desiring men) is one unresolvable shame-producing conflict involving potentially changeable self-standards (e.g., desiring another man is normal/acceptable for me). Changing self-standards might entail changing beliefs about others’ beliefs (e.g., others share and/or accept – rather than reject – me/my desires). Reducing UAI in line with standards (e.g., having sex with another man is normal; I should not risk HIV) may then be easier.

Sex-positive interventions (e.g., accepting, sharing men's same-sex desires as normal) might help MSM differentiate desires and behaviour that need not change (e.g., sex) from self-harmful behaviour that can and must change (e.g., risky sex). To reduce UAI via shame reduction, interventions might: (1) simulate typical sexually and emotionally charged (potentially shame-activating) situations where participants could choose sexual risks (or not), (2) interrupt and unpack affect-based, and as neuroscience models of decision-making suggest [17], automatic processes guiding risky decision-making [18] and (3) remain sex-positive (e.g., shared sexual desires as differentiated from self-harmful choices such as UAI).

Such interventions would be challenging using traditional one-on-one or group interventions. Thus, using an approach called Socially Optimized Learning in Virtual Environments (SOLVE), Miller and her colleagues [19–24] developed and tested interactive, media-based interventions designed to simulate and immerse high-risk young adult MSM in affectively charged risky situations (e.g., an attractive man desiring sex but refusing to use a condom) typically confronted on first dates or “hook-ups.” The player's decisions for his character affect the narrative while learning from virtual mentors/guides and sex partners who accept and share participants’ desires. Throughout the narrative, guide characters (e.g., peers, one's virtual future self) use an ICAP process that involves *(I) interrupting automatic* risky choices, *(C) challenging* those choices with persuasive messages, *(A) acknowledging, accepting and sharing* MSM's emotions/motives (e.g., desires for men) and *(P) providing* a way and skills for MSM to be safe [19–24]. Two prior SOLVE randomized controlled trials (RCTs) demonstrated UAI risk reduction over time. In one trial, at an HIV testing site, HIV-negative MSM exposed to SOLVE (human actors with CD-ROM technology) versus controls (only receiving post HIV-negative counselling) had lower levels of UAI after 10 weeks [21]. In a second, younger (aged 18–24) MSM exposed to SOLVE (interactive DVD with human actors) versus a wait-list control (WLC) condition had lower levels of UAI after three months [19, 20, 24].

Left unclear, however, was whether SOLVE interventions actually reduce shame as intended. To test this, our team, funded by a grant from the National Institute of Mental Health (NIMH), developed an intervention with virtual intelligent agents that incorporated this SOLVE approach into a 3-D animated serious game. Our first hypothesis is that MSM reporting more shame at baseline will report more UAI over the past three months. We also hypothesize that MSM exposed to SOLVE will report immediate shame reductions compared to a WLC condition, and that this shame reduction will predict change in UAI over three months. Finally, shame will mediate the link between condition and UAI change.

## Methods

### Trial design

This online RCT tested the effectiveness of SOLVE, a downloadable simulation video game, compared to a WLC condition in reducing shame and directly or indirectly (via shame reduction) reducing UAI over three months. Randomization was imbalanced [2:1] to compensate for differential loss of participants in the SOLVE treatment condition due to unaddressable technical issues identified in pre-trial piloting (see limitations for details). Online data collection software (Qualtrics) automatically generated the random allocation sequence and assigned participants to condition. Researchers and staff were blind to condition assignment at enrolment, but some were subsequently unblinded to prohibit participant re-enrolment. A data analysis plan was consistently used within and across conditions (addressing out-of-range, missing values; data reduction procedures; outliers, missingness; statistical assumption checks).

### Participants

These data come from the 935 MSM who enrolled between February and November 2012. In this primary prevention intervention, participants were eligible only if they self-reported that they: (1) had a prior HIV-negative test result; (2) lived in the United States; (3) were between 18 and 24 years of age; and (4) engaged in UAI with a non-primary male partner during the three-month period prior to enrolment. We defined a non-primary partner as a man with whom the participant was not currently in a romantic relationship. We targeted young adult MSM because of this group's considerable impact on the epidemiology of HIV in the United States; from 2006 to 2009, estimated HIV incidence increased significantly among MSM only [1]. Because of budget limitations, characters of only three racial groups could be developed for the game. Since the groups most at risk for HIV were Black/African American, Hispanic/Latino or White/Caucasian [1], participants needed to self-identify as belonging to one of these groups. Exclusion criteria included participation in prior SOLVE studies, non-corrected vision/hearing impairment and a history of injecting non-prescribed drugs. Additional exclusion criteria after allocation (resulting in disenrollment and study discontinuation) included not completing baseline measures (since data would have been unanalyzable) and, in the SOLVE treatment condition, being unwilling or unable to download the game and/or being unable to play at least one of two game levels. To recruit, we posted clickable banner ads on websites frequented by the target population. Participants could enter a lottery drawing at baseline with a 1:40 chance of receiving a $100 gift card. At three-month follow-up, participants were offered a $25 gift card.

### Description of SOLVE intervention and control condition

**Figure 1:**
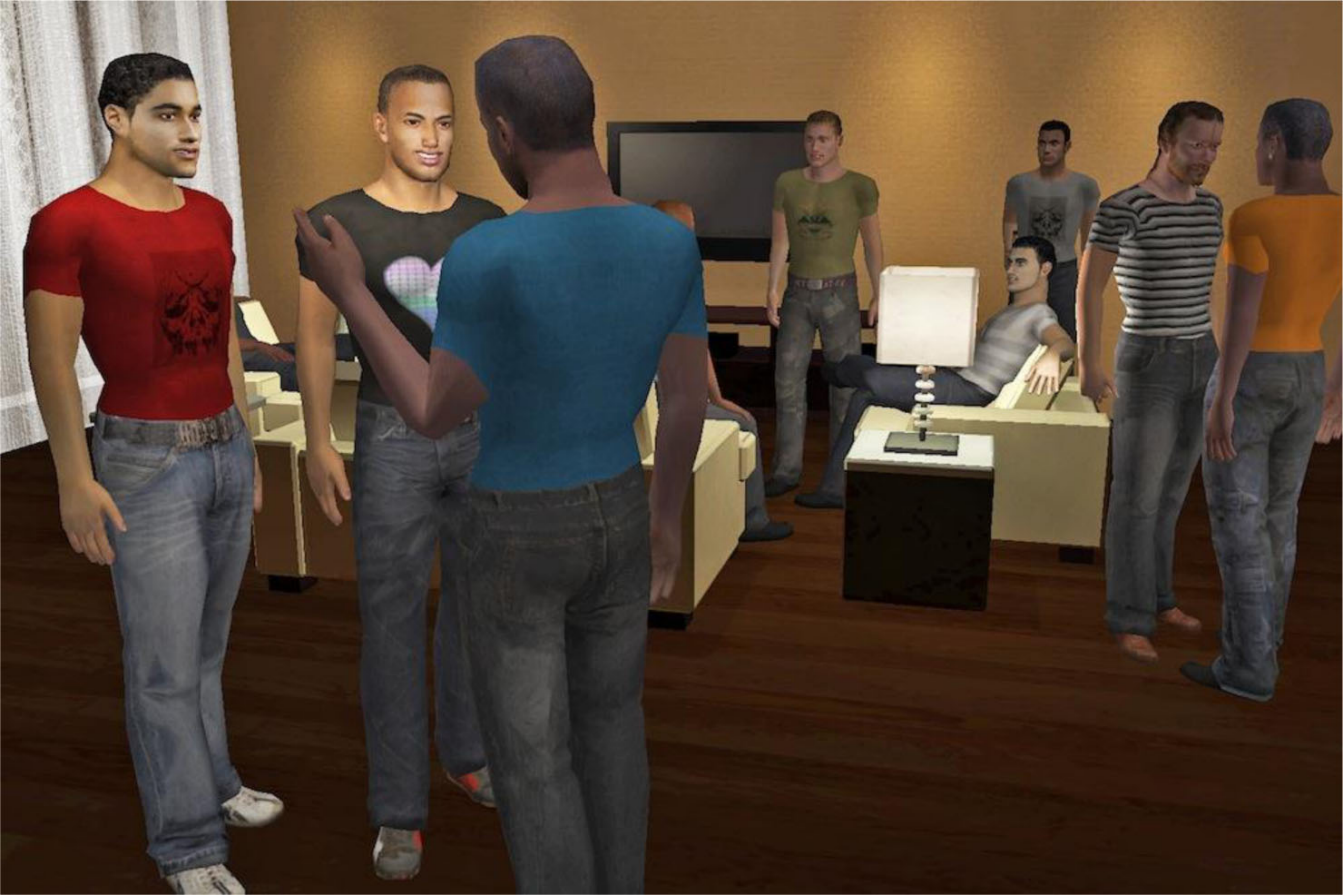
Agents at a virtual house party.

The SOLVE intervention immerses MSM in a virtual world simulating many common obstacles to safer sex. The intervention is guided by cognition-based approaches such as the Theory of Planned Behaviour [25] and Social Cognitive Theory [26] while also capitalizing upon recent advances in neuroscience that suggest emotions are critical during decision-making [17, 18]. The interactive narrative begins after the player customizes his avatar's hair colour, skin tone and clothing style. On the first level, the player can flirt with potential sex partners at a virtual house party (see Figure 1). As the drama unfolds, the player encounters a series of choice points where he must make self-regulatory decisions (e.g., accept/decline multiple offers of alcohol and casual sex). Next, he is at a potential sex partner's apartment. Here, the player gains experience initiating a conversation about safe sex, negotiating condom use and refusing sex if a condom is unavailable. When the player makes a risky choice, he is immediately exposed to a contextualized ICAP intervention. After choosing to engage (or not) in virtual sex, there is a tailored recap sequence where the player's virtual behaviour is evaluated and linked to real-life consequences. Players then move to level two – a virtual nightclub – where the artificially intelligent characters and decision points are more challenging. A primary goal of the intervention is to reduce shame associated with sexual stigma by enabling MSM to more consciously acknowledge their desires and to recognize that their desires are normal. This is achieved through careful design of the characters, dialogue and storylines. For example, the player's avatar consistently models positive self-appraisals and comfort with his sexuality/desires. Through conversations with other characters, he is exposed to dialogue designed to decrease feelings of isolation and inferiority while increasing self-worth. In addition, messages providing HIV knowledge and risk-reduction skills are written/delivered in a non-judgmental, gay-positive manner. Negative feelings associated with religious, societal and familial rejection are also addressed. Participants in the control condition completed the same baseline and immediate post-test measures as those in the SOLVE treatment condition but did not play the game at this time.

### Measures

Consistent with the trial registry, our primary outcome was change in counts of risky sexual behaviour over three months and our secondary outcome was change in shame from baseline to immediate post-test.

#### Risky sexual behaviour

At baseline, participants reported the number of times they engaged in UAI with non-primary partners during the past three-months (i.e., receptive and insertive anal sex without a condom). UAI was reassessed at three-month follow-up.

#### Shame

We measured shame immediately before and immediately after the intervention (or WLC waiting period) using five items from an existing subscale of Watson and Clark's (1994) Positive and Negative Affect Schedule – Expanded Form designed to assess state shame [27]: *ashamed, blameworthy, angry at self, disgusted with self and dissatisfied with self*. Participants indicated how they felt at the present moment using a 1 (*very slightly or not at all*) to 5 (*extremely*) scale. Responses were averaged; internal consistency was high (baseline *α*=0.86; immediate post-test *α*=0.90).

### Ethical considerations

This RCT was approved by the University of Southern California's institutional review board (IRB). To ensure confidentiality, participants were only identified via email address, which was deleted upon study completion.

### Data analysis

Of the 935 participants, 14 (seven per condition) “completed” their baseline measures but responded to each shame item with “refuse to answer” and therefore could not be included in the main analyses. Data from 921 MSM (control condition, 484; SOLVE treatment condition, 437) were analyzed.

Using the mean function in SPSS-20, we replaced missing values on the five baseline shame items with the mean of values present for that participant. We then calculated a shame change score. Simple difference scores are typically correlated with baseline values and so we used residualized change scores, which eliminates this dependency [28, 29]. Residuals for shame were calculated by regressing immediate post-test values (Y) on baseline values (X), affording estimates of predicted Y values (Y’) that are then subtracted from Y (and saved as unstandardized residuals). Positive scores indicate an increase; negative scores a decrease. Where a residualized score could not be calculated due to completely missing immediate post-test data, we used the average residual of participants with matching baseline shame scores. Change in UAI was computed by regressing three-month follow-up values on baseline values. UAI change scores of those lost to follow-up were estimated in M*Plus* 7 using a full-information maximum likelihood (FIML) procedure [30].

To test whether prior UAI predicts baseline shame, we used the Spearman correlation coefficient because of the positive skew that is typical of count variables such as UAI (*z*=30.0; *p*<0.001). An independent-samples *t*-test examined whether shame was reduced in the SOLVE treatment condition versus control condition. Tests are reported as two-tailed and a *p*-value of 0.05 indicates statistical significance. To test the proposed mediation model, we used the “Model Indirect” command within M*Plus* 7 [30]. Bias-corrected 95% confidence intervals were generated using 5000 bootstrap samples. An indirect effect is observed if the confidence interval is entirely above or below zero. Using a Mahalanobis distance critical value of 13.8, we detected and removed three multivariate outliers in the WLC and five in the SOLVE treatment condition.

## Results

**Figure 2:**
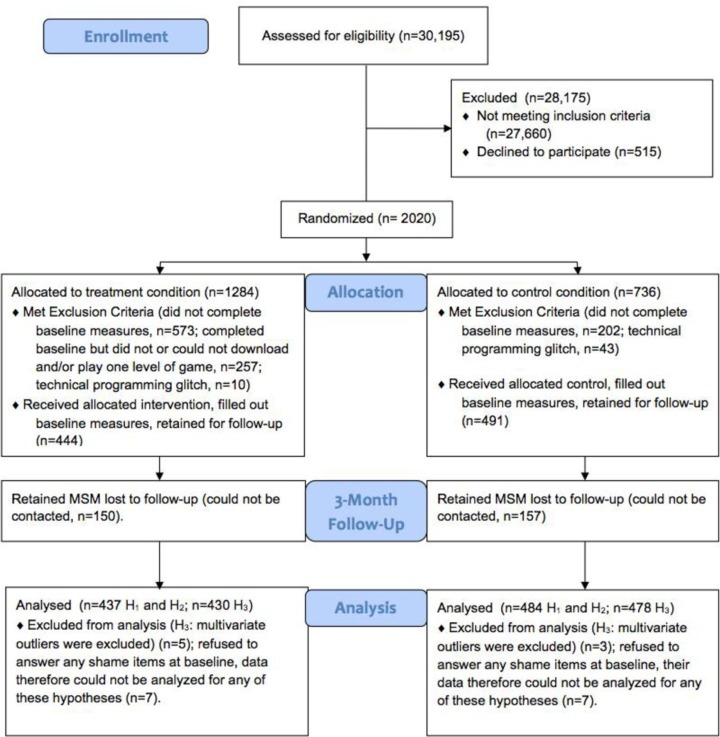
Participant flow diagram comparing the SOLVE treatment condition and control condition.

The CONSORT [31, 32] diagram of participant flow presented in Figure 2 provides information regarding the number of participants assessed for eligibility, randomized to condition, lost to follow-up and included in the main analyses. Of those receiving the allocated intervention or control, 73% were White/Caucasian, 14% were Latino/Hispanic and 13% were Black/African American. The majority identified as gay or homosexual (76%) and had at least some post-secondary training (82%). Approximately 13% reported living in a rural geographic area. Participants were 21 years old on average, had engaged in UAI about 12 times in the past three months and reported relatively low levels of baseline shame (1.7 on a five-point scale). See Table 1 for measures split by condition. A randomization check confirmed that the conditions did not significantly differ on any of the baseline measures. Consistent with past work [19–21, 24], no ethnic differences for any analyses were found.

**Table 1:** Baseline characteristics by condition

	Control (*N=*491)	Treatment (*N=*444)	Difference by condition
Race/ethnicity			*p*=0.77
White/Caucasian	349 (71.1%)	338 (76.1%)	
Latino/Hispanic	76 (15.5%)	55 (12.4%)	
Black/African American	66 (13.4%)	51 (11.5%)	
Sexual orientation			*p*=0.49
Gay/homosexual	376 (76.6%)	331 (74.5%)	
Bisexual	56 (11.4%)	65 (14.7%)	
Other	57 (11.6%)	46 (10.4%)	
Postsecondary education	394 (80.2%)	371 (83.6%)	*p*=0.48
Rural geographic area	66 (13.4%)	57 (12.8%)	*p*=0.74
Age (mean, SD)	21.3 (1.8)	21.3 (1.7)	*p*=0.73
UAI (mean, SD)	12.5 (11.8)	11.7 (12.5)	*p*=0.32
Shame (mean, SD)	1.7 (0.82)	1.7 (0.77)	*p*=0.54

### Main analyses

Risky sexual behaviour and shame. As predicted, we found that prior sexual risk-taking was positively correlated with baseline shame, *r*_s_=0.21, *p<*0.001, 95% CI [0.15–0.27].

#### Change in shame due to the SOLVE treatment versus control condition

Exposure to the intervention led to immediate mean shame reduction for those in the SOLVE treatment condition (*M*=−0.08, *SD*=0.51, *n*=437) while it unexpectedly led to an increase (*M*=0.07, *SD*=0.54, *n=*484) in the control condition (Table 2). The difference was statistically significant, *t*(919)=4.24, *p<*0.001. Cohen's *d*=0.29.

#### Mediation analysis

We used a bootstrapping approach to assess the effect of condition on UAI change indirectly through shame change. As expected, condition predicted shame change (path a), *B=*−0.14, SE=0.03, 95% bias-corrected CI [−0.07 to −0.20], and shame change predicted UAI change (path b), *B=*0.73, SE=0.36, 95% bias-corrected CI [0.03–1.45]. The direct effect of the intervention on UAI change was not significant but, as hypothesized, the indirect effect was negative and statistically different from zero; point estimate=−0.10, 95% bias-corrected CI [−0.01 to −0.23]. Participants in the SOLVE treatment condition reported greater reductions in shame, which in turn influenced reductions in risky sexual behaviour at follow-up.

**Table 2:** Means for shame items

	Control		Treatment	
		
	Pre-test	Post-test	Pre-test	Post-test
Ashamed	1.70	1.73	1.66	1.55
Blameworthy	1.69	1.71	1.60	1.60
Angry at self	1.68	1.70	1.70	1.58
Disgusted with self	1.73	1.63	1.57	1.49
Dissatisfied with self	1.84	1.91	1.93	1.70
Scale total	1.73	1.74	1.69	1.58

## Discussion

Our *a priori* hypothesis that a web-based simulation game would reduce sexual shame was supported. This reduction, in turn, indirectly reduced UAI following the intervention. These findings are exciting because they suggest that, for some MSM, shame reduction may be an important intervention component resulting in UAI change. Condition, however, did not have a direct effect on UAI in this study, as it had in two prior SOLVE studies. This SOLVE intervention differed from earlier versions in a number of ways (e.g., animated versus life actors; national sample versus Los Angeles; participation over the web under “real-life” and less controlled conditions). We are currently exploring potential suppressors of the link between condition and UAI that might have resulted in an overall insignificant direct effect [33]. Such mediational analyses are critical in identifying what works (and does not) for whom, to better optimize and cumulatively advance our risk-reduction interventions. Additional analyses with this sample indicated that measured fear was not predictive of risk-reduction, indicating that shame specifically matters, and not negative emotions generally.

To our knowledge, this is the first HIV prevention intervention to demonstrate shame reduction. Although we did not expect shame to change in the control condition, a slight increase was observed. Might increased shame be a by-product of recently reporting and ruminating about one's prior risky behaviour? Future research should examine how survey items may negatively affect participants.

SOLVE is one of a family of recent theory-based interventions to reduce shame among high-risk individuals [34]. These interventions share a focus on greater self-awareness of emotions, goals, behaviours and associated barriers while fostering acceptance of parts of the self that cannot change. SOLVE's focus on counselling and social support can be compared to several intervention efforts aimed at reducing HIV stigma. A recent systematic review by Sengupta and colleagues identified four interventions that attempted to reduce HIV stigma using this approach [35]. The duration of these interventions ranged from six hours to one year. SOLVE, in comparison, is brief (e.g., 30 minutes).

SOLVE, unlike most HIV prevention interventions, is completely deliverable over the Internet. In this regard, SOLVE is similar to at least two other recent interventions designed to improve the wellbeing of MSM. In one study, a web-based expressive writing intervention for gay-related stress led to improvements in psychosocial functioning, including increased sexual orientation openness [36]. Another web-based HIV prevention intervention called *Keep it Up!* successfully used videos, animation and games to reduce rates of UAI [37]. Collectively, these interventions demonstrate the plausibility of rapid dissemination and broader reach – with potentially greater cost effectiveness.

### Limitations

Nationwide web-based testing of an intervention is challenging. Glitches internal to the game itself were remedied pre-trial; however, some participants would not download an executable file. Others could not play the game given hardware (e.g., CPU, Internet speed, memory, disk space, graphics card) and software configurations (e.g., operating system age and version; conflicts with other software). Given rapid computer configuration changes, this remains a “moving target.” Although “dummying down” the technology is tempting, two considerations argued against that: (1) prior work indicates that immersion/presence predicts behaviour change [38]; and (2) if effective, the intervention might then have a shorter “shelf-life” after trials conclude. We attempted to use meta-data to identify participants with technical issues but found no discernable predictor pattern. A lesson learned is that a mini-game in the screener might reliably discern if players, randomized to a game condition, could (or would) download/play it. Although it is possible to play the game at a local intervention site or cyber-café, we recommend privacy since the player's virtual choices may be subject to social desirability bias if others are present.

A second limitation was our retention rate (69%). At three-months, this was under the desired cut-off of 70% for “best evidence,” specified by the Centres for Disease Control and Prevention [39]. Although on-line studies are still rare, some researchers have assessed the retention of MSM in RCTs and have found it can be surprisingly low over three months. Across four other studies, the rates were 15% [40], 25% [41], 53% [42] and 95% [43]. Thus, the current study had one of the highest retention rates for online studies over three months with MSM to date.

Third, financial constraints precluded developing characters other than Black, White or Latino, making the game potentially less suitable for other MSM. Even for Black and Latino MSM, finances constrained our ability to include culturally targeted dialogue, pop-culture references and behavioural choices as we had in prior interventions. Nevertheless, no ethnic differences were found in the current work. Financial constraints also limited our ability to develop storylines to address unique issues faced by people living with HIV (PLHIV). In addition to shame as a manifestation of sexual stigma, it is likely that some MSM living with HIV would also be experiencing shame associated with HIV-related stigma and discrimination. Future research should explore how intersecting stigmas might be best addressed in serious games and other technology-enabled interventions for PLHIV. The framework described by Stangl and colleagues may provide a useful starting point [44].

In this national online study, financial, ethical and practical considerations made the collection of bio-markers of risky sexual behaviour infeasible, forcing a reliance on self-report measures alone. Nevertheless, if a game is widely used nationally, other local measures (e.g., of condom sales; STI rates) tied to participant zip codes might provide alternative, inexpensive methods for assessing condom use.

The study is also limited in that participants may have been reluctant to self-report feelings of shame [9], reducing our ability to detect shame reduction. It should be noted that the degree of shame reported by participants was relatively low, corresponding to the anchor labelled “a little.” We are currently investigating possible non self-report methods of shame that could be gathered unobtrusively during a game promising better predictability [14].

### Generalizability

SOLVE's process of game development is designed to enhance generalizability. First, the content was based on several qualitative and quantitative pilot studies that allowed us to understand and map common story arcs, obstacles to safe sex and personal preferences. We sampled MSM across the United States, allowing urban and rural geographic representation. Input from several population-matched community advisory boards throughout informed the design process. Despite successfully recruiting and retaining Black and Latino MSM online, larger sample sizes would afford more granular within-group analyses. Finally, this trial was conducted exclusively online, supporting the feasibility of rapid dissemination and evaluation of serious games (and more traditional interventions) targeting diverse, hard-to-reach high-risk populations.

## Conclusions

Overall, these RCT findings indicate that a game intervention can reduce shame and suggest that such reductions are diagnostic of future reductions in sexual risk-taking for young adult MSM. In ongoing research, we are addressing whether reduction in shame predicts reduction in UAI at longer time intervals (i.e., six months). We also plan to examine which of these components alone or in combination might reduce shame for MSM using more sensitive measures (e.g., neural signals). Another goal for future research is to examine whether a SOLVE game approach can be effectively generalized to other target groups and other risk-reduction efforts. Although technology-based interventions tested and disseminated over the web are promising, understanding how to better adapt these tools for limited resource settings and better overcome the technical challenges posed is critical.
